# Opposite molecular sex correlations in tauopathy paralleled by motor and cognitive efficacy of davunetide in women

**DOI:** 10.1038/s41380-026-03709-x

**Published:** 2026-07-03

**Authors:** Guy Shapira, Jason Blatt, Liri S. Guz, Noam Shomron, Illana Gozes

**Affiliations:** 1 Gray Faculty of Medical and Health Sciences, Sagol School of Neuroscience, Edmond J Safra Center for Bioinformatics, Tel Aviv University, Tel Aviv, 6997801 Israel; 2 The Elton Laboratory for Molecular Neuroendocrinology, Gray Faculty of Medical and Health Sciences, Sagol School of Neuroscience and Adams Super Center for Brain Studies, Tel Aviv University, Tel Aviv, 6997801 Israel

**Keywords:** Diseases, Biomarkers

## Abstract

Progressive supranuclear palsy (PSP) is a fatal tauopathy presenting an unmet medical need. Davunetide, targeting tauopathy, has been previously tested in PSP, which, when separated by sex, implied efficacy in women. Here, efficacy was evaluated using longitudinal data (52 weeks) and applying for the first time, the US Food and Drug Administration (FDA) -recommended 10-item PSP Rating Scale (PSPRS-10) compared to the 28-item PSPRS (primary endpoint). Time-, sex-, and treatment-related effects were analyzed using linear mixed-effects models including the FDA-recommended Mixed Models for Repeated Measures (MMRM). Cognitive evaluations used the Repeatable Battery for the Assessment of Neuropsychological Status (RBANS). Clinical analyses at the individual level accentuated the impact of the 10 PSPRS chosen items and revealed a significant treatment effect favoring women when accounting for time, sex, and treatment interactions (*P* = 0.0005). Motor measurements were highlighted. RBANS analyses demonstrated women’s significant improvements in total raw score, letter–number sequencing, and language scores. Correlating cerebrospinal fluid (CSF) phosphorylated Tau/Tau with functional outcomes revealed significance with p-Tau/Tau ratio and grasping/imitative/utilizing behavior (assessing involuntary actions in PSPRS-28). This correlation was opposite in direction between females (r = 0.77; *P* = 0.006) and males (r = -0.62; *P* = 0.075), with sex difference showing a *P* = 0.0002 value. Similar results were obtained for language. Predicting the final PSPRS score (week 52), addressing all clinical measures, a clear woman-treatment-specific pattern in outcome importance was discovered, highlighting significance for the primary endpoint Schwab and England Activity of Daily Living (SEADL) scale, and stressing the necessity for sex-specific medicine with davunetide as a lead compound.

## Introduction

Widely ignored in previous clinical research are the innate molecular differences between males and females, which may strongly influence clinical outcomes. While studies in the most prevalent tauopathy, Alzheimer’s disease (AD), showing a significantly larger women patient prevalence, are now accumulating [1], the rare pure tauopathies, like progressive supranuclear palsy (PSP), are evaluated to a much lesser degree for sex differences associated with microtubule/Tau pathology. However, animal studies strongly suggest sex-specificity for pure tauopathies [2–6]. With tauopathies playing a central role in neurodegenerative as well as neurodevelopmental diseases [7, 8], the sex-dependent aspect in the clinical scenario needs to be further pursued.

Studying vasoactive intestinal peptide (VIP), a central neuropeptide protective in AD rodent models [9–11] for sex specificity under ovary or orchidectomy, revealed a decrease in hypothalamic *VIP* mRNA expression post-ovariectomy, and no change in *VIP* levels following orchidectomy. With the introduction of estrogen replacement, the decline was reversed. This demonstrated a clear estrogen-dependent regulatory mechanism, highlighting vital differences in sex-specific neuroendocrine gene expression, predicting more recent findings [12].

Sex-dependent treatment has been a further focus of our research, analyzing the VIP regulated activity-dependent neuroprotective protein (ADNP) [6, 10]. Discovered in our laboratory [13], ADNP is essential in brain formation [14] and function, with a neuroprotective active site (i.e., the investigational drug davunetide, also known as NAP, AL-108, CP201, and NAPVSIPQ), clinically evaluated for neuroprotection in several chronic and acute brain diseases associated with impaired neuronal function and neurodegeneration [10, 15].

Involving cytoskeletal and autophagy protection that is aberrant in schizophrenia and neurodegenerative diseases [16], davunetide (NAPVSIPQ) has been shown to implicate neuroprotective activity. Furthermore, a point mutation in NAPVSIPQQ to NAPVSIPQE in ADNP caused a developmental syndrome affecting muscle, feeding, and social behavior of the afflicted child [17]. Mechanistically, by associating with microtubule end-binding proteins (EB1/EB3) via the SxIP motif in NAPVSIPQ, it enhances microtubule dynamics and Tau-microtubule interactions [18, 19], thus protecting against tauopathy in a sex-dependent manner while facilitating dendritic spine formation [5]. Importantly, the entire NAPVSIPQ sequence further regulates key pathways, including Akt [5, 20], Wnt [17], and SH3-linked signaling [the NAPVSIP motif [21]], complementing microtubule/cytoskeletal protection.

As indicated above, our laboratory has previously associated AD Tau pathology and sex-dependent davunetide corrective effects [10, 22]. More specifically, we discovered somatic mutations in ADNP in postmortem brains of old subjects and AD subjects, correlated with increased tauopathy [8]. Additionally, in a postmortem analysis of a boy’s brain carrying a heterozygous de novo pathogenic ADNP mutation (inflicting intellectual disabilities/cognitive impairment/autistic traits), extensive tauopathy was observed [7]. This is consistent with our animal studies modeling ADNP mutations in mice [3, 5, 20, 23], showing increased tauopathy in young male mice, compared to female mice [5, 24], which is ameliorated by davunetide treatment. These findings are coupled to age-dependent regulation of gene expression by ADNP [3, 20], setting the stage for age/sex-dependent davunetide protection in humans. Notably, tying back to VIP sexual dichotomy, we discovered estrous cycle regulation of *ADNP* mRNA in the hypothalamus, cyclic fluctuation of ADNP immunostaining in the arcuate nucleus in female mice [25] and ADNP regulation of steroid biosynthesis genes [7].

Clinically, in our 2023 study entitled “Unexpected gender differences in progressive supranuclear palsy reveal efficacy for davunetide in women,” sex-dependent treatment response and disease progression were highlighted [26]. Analyzing PSP patients, compared to males, female subjects showed faster deterioration, greater brain ventricular volume increase compared to baseline measurements, and efficacious treatment response, with twice-daily davunetide-treated female subjects showing slowed disease progression, with improved motor skills. In this respect, it should be noted that any exposure to estrogen replacement therapy halved the risk of PSP in women (odds ratio = 0.52; 95% confidence interval = 0.30-0.92; P = .03). Among PSP cases, earlier age at menarche was associated with better performance [27]. Most importantly, in people exhibiting preclinical AD, tauopathy progression in the brain is sex-dependent [1]. Furthermore, a recent elegant study showed that lower Repeatable Battery for the Assessment of Neuropsychological Status (RBANS)-language at baseline predicted greater PSP Rating Scale (PSPRS) worsening over time. The low language-score group showed poorer cognitive performance, increased circulating neurofilaments and reduced gray matter volume in language-related areas [28]. In this respect, our mouse findings indicate ADNP regulation of mouse vocalizations [20] and syntax [5] in a sex-dependent manner, paralleling sexual dichotomy in the regulation of dendritic spine formation and tauopathy [5]. These findings, touching the tip of the iceberg, highlight the need for further sex-specific analysis on drug efficacy, neuroprotection, and molecular/cellular neurodegeneration. Thus, the requirement to extend our research to understanding sex-specific drug molecular translatability to efficacy is evident.

Focusing on PSP, presenting a neurodegenerative disorder impacting movement and cognition, it is of further interest to dive deeper into the pathogenesis of the disease. PSP tauopathy is caused by abnormal Tau aggregation with 4-repeat (4 R) microtubule binding domains encoded by the Tau gene (*MAPT*), which contains recurrent genomic inversion (presented by H1 and H2 haplotypes). In a recent Tunisian PSP study, phenotypes were categorized into three subgroups: Richardson’s syndrome (PSP-RS, 44.06%), PSP-cortical (31.35%), and PSP-subcortical (24.57%). Regarding the *MAPT* genotype, significant female predominance was observed among H1/H1 carriers. The findings suggested that PSP is linked to haplotype-dependent increases in 4 R Tau mRNA, which contributes to the pathogenesis of PSP and influences its clinical phenotype. Homozygous H1 carriers developed Parkinsonism and oculomotor signs earlier, more frequent freezing of gait, and vertical supranuclear gaze palsy. The homozygous *MAPT* genotype was further associated with cognitive (memory, language) impairment than the heterozygous profile [29].

Given the differences amongst PSP subtypes, we concentrated here on PSP-RS patients [30]. 95% of the cohort presented the H1 haplotype, while the remaining participants were H1/H2 (20% of the participants were not genotyped) [30]. Separating the H1/H2 population in our cohort into males and females showed male predominance in the H1/H2 genotype (about 62%) in agreement with the cited Tunisian cohort [29]. Importantly, our biomarker cohort did not include any individuals presenting an H1/H2 genotype, toward precision medicine.

## Methods

### Davunetide in treatment of progressive supranuclear palsy (PSP) Richardson syndrome: study population

In a 52-week Phase 2/3, randomized, double-blind, placebo-controlled study, the efficacy and safety of intranasal davunetide 30 mg BID relative to placebo in treating PSP were evaluated. Primary endpoints for efficacy included change from baseline scores of the PSPRS at 52 weeks and change from baseline of the Schwab and England Activities of Daily Living Scale (SEADL) at 52 weeks. Safety was measured by reported adverse events, electrocardiograms, nasal examinations, and clinical laboratory measures [26, 30]. 313 participants (aged mid-40s to mid-80s) were randomly assigned to davunetide (*N*  =  157) or placebo (*N*  =  156) groups. Of these, 241 (77%) completed the study (118 and 123 in the davunetide and placebo groups, respectively) [30]. The clinical study report (CSR, NCT01110720) transferred to us included 312 subjects, some with most or all data, others with very little data, as detailed below.

This NCT01110720 trial testing davunetide was originally sponsored by Allon Therapeutics assuming no sex differences in PSP. The study results were published, led by the study PI, Adam Boxer, concluding that davunetide is safe and well tolerated, but not showing efficacy in PSP [30]. The davunetide technology was then requested and returned to Ramot (patents holder) at Tel Aviv University and is now licensed to ExoNavis Therapeutics Ltd. Based on accumulating preclinical data indicative of dramatic, highly significant sex differences in ADNP/davunetide neuroprotection [3, 5, 6], we, Illana Gozes, davunetide & ADNP inventor, Tel Aviv University, Allon Therapeutics’ Co-Founder, Board Member & Chief Scientific Officer, ExoNavis’ Vice President for Drug Development, reanalyzed the Allon statistical data files separated to men/women populations together with Guy Shapira, Alexandra Lobyntseva and Noam Shomron [26]. As stated in our published study [26], “analyzing sex-dependency, demonstrated baseline ventricular volume-dependent/volume increase correlations in 52-week-placebo-treated women (r = 0.74, *P* = 2.36^-9^), whereas davunetide-treated women (like men) revealed no such effects. Primary endpoints, highlighted by the SEADL scale, showed significantly faster deterioration in females, starting at trial week 13 (correlating with most other endpoints by week 52). Davunetide slowed disease progression and revealed significant protection according to the SEADL scale as early as at 39 weeks, as well as protection of the bulbar and limb motor domains considered by the PSPRS, including speaking and swallowing difficulties caused by brain damage, and deterioration of fine motor skills, respectably, at 52 weeks”.

However, the original study [30] as well as the reanalysis, separating the sexes [26], used the 28 items of assessment PSPRS, and the FDA currently requires the more specific 10 items, PSPRS-10^31^. Thus, for further in-depth evaluation of PSPRS-10 and assessment of the individual items on PSPRS-28, we reverted from the CSR measurements to the utilization of the available individual Clinical Report Forms (CRFs), extracting the 28 PSPRS items. The PSPRS score was reanalyzed using the 10-item version with the sum of item scores as the independent variable [31]. In short, we used the recent PSPRS-10, which is now the FDA-approved standard scale, featuring improved measurement efficiency, statistical power, and sensitivity [32, 33].

Given the recent findings of RBANS language at baseline predicting greater PSPRS worsening over time and associated with brain pathology [28] were in-depth measurements of efficacy by the exploratory endpoints (detected but not elaborated on before) [26]. These included change from baseline of cognition by the RBANS, and Executive Function Tests at 26 and 52 weeks. The RBANS is a validated mechanism that assesses 5 cognitive domains: Immediate Memory, Delayed Memory, Attention, Language, and Visuospatial/Constructional [34]. The Executive Function Tests measure Phonemic Fluency, the Color Trails Test, and Letter-Number Sequencing.

With PSP being a leading neurodegenerative tauopathy, we further correlated, for the first time, all study outcomes [26, 30, 35] with Tau, phosphorylated Tau 181 (p-Tau), and neurofilament light chain (NFL) cerebrospinal fluid (CSF) concentrations, after lumbar puncture. Plasma was collected for assessment of phosphorylated neurofilament heavy chain (pNFH), at baseline and 52 weeks. As indicated, all values obtained in 11 females (3 davunetide-treated and 8 placebo) and 13 males (8 davunetide-treated and 5 placebo-treated, available to us) were correlated, separating males and females.

### Statistical methods

#### Overall statement

Our comprehensive statistical testing (below) brought up multiple, seemingly correlated measurements in the results. This coordinated change is, to some extent, an expected result of internally consistent improvement or worsening of disease severity. Within the framework of the clinical trial, this multiplicity is prevented by defining strict primary endpoints ahead of the trial. In this ad-hoc analysis, the co-primary endpoint (PSPRS) remained our focus, but the rigidity of the original analysis was no longer relevant. As with other post-hoc analyses of clinical trials, we acknowledged the generality of change in outcome measurements including cognitive endpoints, which could not be confidently attributed to a single independent domain [36].

#### Mixed models for repeated measures (MMRM)

MMRM is a type of model that is commonly used for analysis of PSPRS scores and longitudinal clinical data in general (gold standard) [33]. Models were created using R’s MMRM package and used for per-timepoint comparisons. Additional models were made in the lmerTest package, which was used for confirmation and continuous timepoint modelling (it also features different optimizers, random effects handling, etc.). Post-hoc estimation of marginal means and testing of simple effects from the full model were performed using the emmeans package and visualized using the ggeffects package and based on the continuous model.

To model score difference from baseline, we used the design:

diff Score ∼ baseScore + timepoint ∗ Sex ∗ Treatment + (1 + timepoint|ID)

Several formulas were tested however the current choice was based on fitness metrics (AIC/BIC) and likelihood ratio tests.

Additional statistical analysis was performed in R, version 4.2.1 (2022-06-23) with *rstatix* 0.7.2 and visualized with ggplot2-based figures. Basic tests utilized Pearson correlation and pairwise Wilcoxon as previously described [26]. A generalized linear mixed model (GLMM) was fitted with timepoint, sex, and treatment as dependent variables (interactions included). The Poisson distribution was chosen for the response variable, as it is better suited for non-negative integers and provided a better fit. Notably, predictive modelling was performed using a generalized Poisson model trained on the patient’s baseline properties, with the final (week 52) PSPRS score as the outcome variable. Feature importance was determined from the standardized coefficients of this model, derived by the VIP R package (v0.4.5) [37].

Further analyses used GraphPad Prism 10.2.3 software included D’Agostino and Pearson tests for normality, coupled with log10 transformation to achieve normal distribution. A two-way analysis of variance (ANOVA) followed by post hoc Tukey’s Honest Significant Difference (HSD) test was then used to assess significant differences. Both Wilcoxon two-tailed and unpaired two-tailed t-tests were utilized RBANS, and Executive Function Tests.

Noteworthy, the original PSP results [30] were independently analyzed separating men and women [26], and verified by Bio-statistic | Medistat Ltd | Tel Aviv District, Israel, as well as DataSights - From Data to Insights, Israel (supported by ExoNavis Therapeutics Ltd., holding a license for the davunetide technology).

#### Power analysis

To calculate statistical power, we used the longpower package [38] (approximated by the power.mmrm function, to avoid the non-linear simulation), with the 6-months minimal clinically important change of 5.7, in accordance with Hewer, et al., [39]. The power of the female only comparison was approximately 0.8.

## Results

### PSPRS individual assessments indicative of functional degeneration and woman-specific protection

**Fig. 1:**
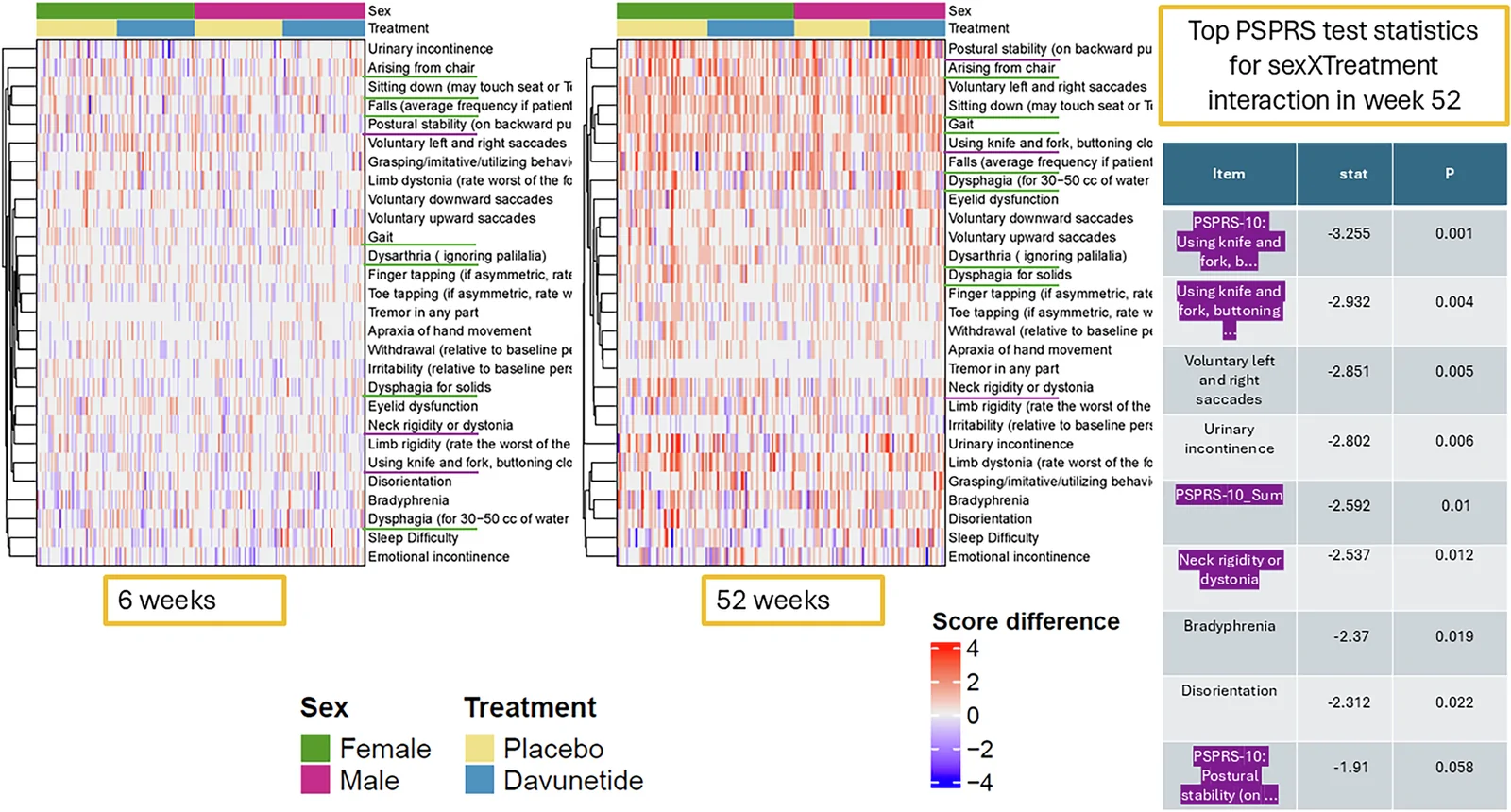
Disease Time Progression (52 Weeks) Per Patient: Protection by Davunetide in Women and Increasing Effects on the FDA-Recommended PSPRS-10 Individual Outcome Measures. Heatmap visualization of the 28 outcome measures of the PSPRS score, with PSPRS 10 outcome measures underlined in green or violet (Table), clustering the difference from baseline at 6 weeks and 52 weeks of twice daily davunetide applications (30 mg/dose). The number of individuals with measurable data is depicted in Table S1, further including individuals with some but not all measurable items (an additional man for placebo and for davunetide as well as an additional woman placebo, 4 davunetide-treated women at time 0 and one additional davunetide-treated male at week 52). The Table shows top PSPRS test statistics for sexXTreatment interaction in week 52, PSPRS-10: Using knife and fork, b… = knife and fork use, buttoning clothes, washing hands and face; and PSPRS-10: Postural stability on… (backward pull) refers to data re-calculation using only PSPRS-10 (P ≤ 0.058) items.

Toward precision medicine, we first looked at the individual level, comparing sex/drug effects after 6 and 52 weeks of twice daily davunetide applications (30 mg/dose). Results (Fig. 1) depict disease time progression per patient utilizing a heatmap visualization of the 28 outcome measure items of the PSPRS score, with PSPRS-10 outcome measure items underlined in green (or violet, implying most significant davunetide protection). Visual inspection indicated an overall deterioration spanning the 52-week-treatment, with lesser deterioration in the davunetide-treated female group. Looking at the impact of the 10 different item measurements of the PSPRS-10 and comparing 6 to 52 weeks showed increased impact for the PSPRS-10 items over the excluded PSPRS-28 individual measurements, for example, for postural stability. Specifically addressing the individual items using MMRM comparisons indicated a *P* < 0.005 for using knife and fork (considering the either the 28 PSPRS items, or the 10 PSPRS items of significance, rescaled, Fig. 1, Table). Significant results were also seen for the entire PSPRS-10 and for neck rigidity or dystonia, while postural stability was almost significant (*P* = 0.058, rescaled, Fig. 1 Table shows PSPRS test statistics for sexXTreatment interaction in week 52).

### Significant protection for davunetide in women using PSPRS-10

**Fig. 2:**
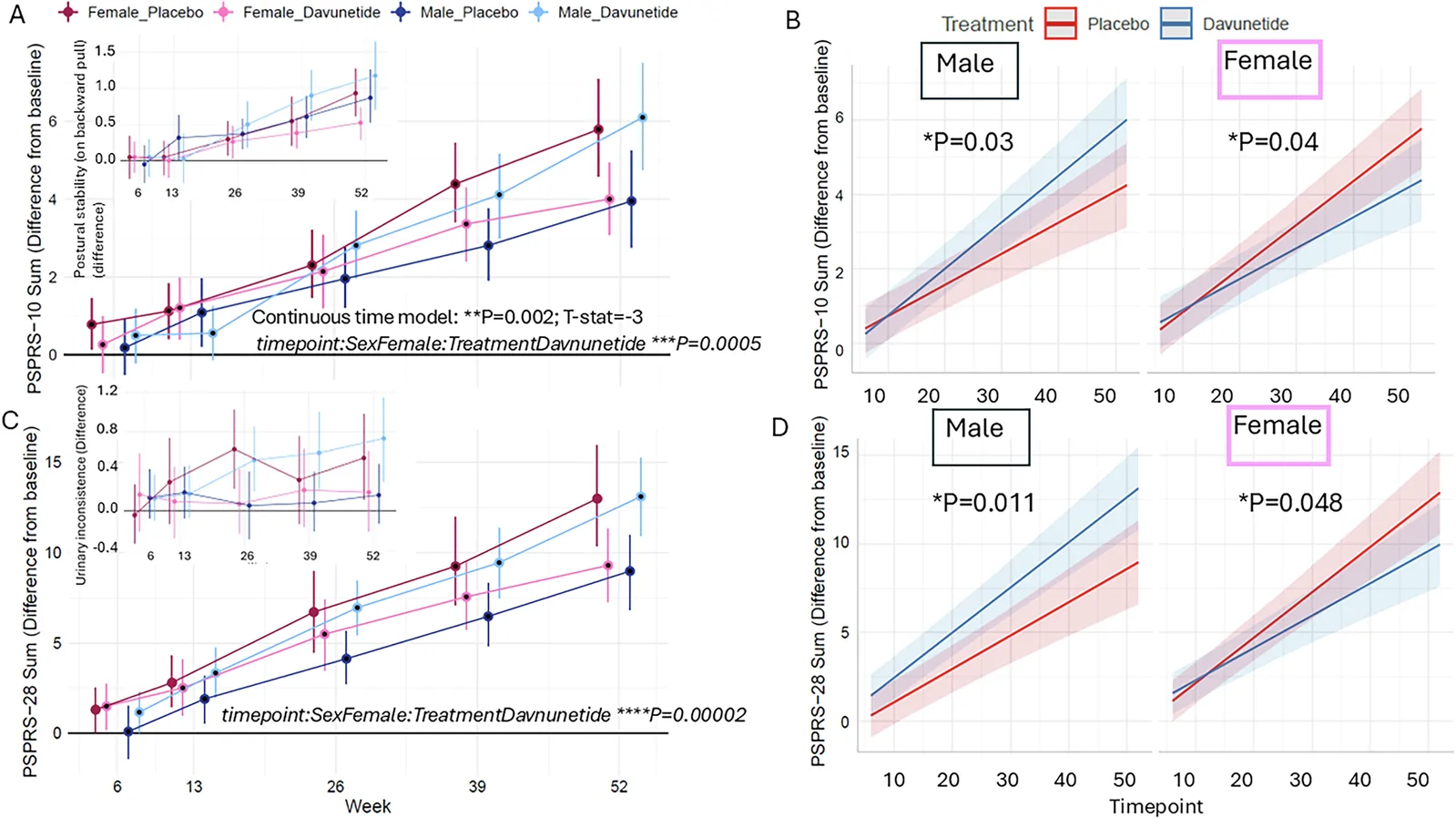
Woman Specific Davunetide Efficacy in PSPRS-10. Table S1 indicates the number of patients included in the PSPRS-10 MMRM analysis (for the **A** and **B** panels), and Table S3 shows the number of patients included in the PSPRS-28 MMRM analysis (**C** and **D** panels). **A** Results show a comparison of the four experimental groups for PSPRS-10 using the “SumS” method [31] across 5 timepoints. Insert depicts postural stability, which at the individual patient was most affected at 52 weeks (Fig. 1). **B** Marginal means for summarized PSPRS-10 by treatment and sex. **C** Results show a comparison of the four experimental groups for PSPRS-28 across 5 timepoints and compared to the PSPRS-28. Insert depicts urinary incontinence, which at the individual patient was most affected at 6 weeks (Fig. 1). **D** Marginal means for summarized PSPRS-28 by treatment and sex. Significance is indicated as per MMRM results (Tables S2 and S4) and italics for GLMM analysis.

The summarized PSPRS-10 was modeled in response to timepoint (weeks from baseline), sex, treatment and the interaction between the three (MMRM). Tests compared davunetide treatment to placebo for each sex and the sex-dependent difference in davunetide response (SexXTreatment interaction (Fig. 2A, B). Table S1 depicts the precise number of individuals participating per tested group (ranging between *N* = 46 to *N* = 60, having all PSPRS measurements per time point).

The full model indicates that PSPRS-10 significantly decreased for davunetide-treated females relative to the effects in males (Continuous time model: *P* = 0.002; T-stat = -3, Fig. 2A). We further examined the underlying per-sex and per-timepoint effects to better understand this result (Table S2). There was an initial sex-specific difference, driven by sub-significant effects in both sexes and curbed in the next timepoint. At week 39, we saw a trend of male PSPRS-10 increase and the opposite in females. By week 52, the male and female score trends became individually significant, as well as significantly diverging in a sex-dependent manner, with davunetide efficacy in women (Fig. 2B, *P* < 0.05).

Furthermore, sex-treatment interaction over time (Sex:Treatment:Timepoint Significance) was ruled significant according to GLMM, applying to PSPRS-10 Sum (*P* = *0.0005*; Fig. 2A). Figure 2A insert shows the time/sex/treatment graph for postural instability paralleling the PSPRS-10 findings (Fig. 1 Table).

The summarized score for the original, 28-item PSPRS was available for more participants (CSR), rather than the collected original individual CRFs (hence the separation) (CSR, Table S3, N range, 55-71). Results for PSPRS-28 (Fig. 2C, D), calculated as in Fig. 2A, B (Tables S4 and S2, respectively), showed similar significance (*P* < 0.05). Furthermore, sex-treatment interaction over time was ruled significant according to GLMM, also applying to PSPRS-28 (*P* = *0.000018*; Fig. 2C).

As indicated above, Fig. 1 Table insert shows the top PSPRS test statistics for sexXTreatment interaction in week 52. An assessment of each of the 28 PSPRS items with an ordinal version of the same sex:treatment:timepoint interaction model, found significant results for urinary incontinence (*P* = 0.0016). Interestingly, this significant item is excluded from the PSPRS-10 scale [31] (Fig. 1), regardless urinary incontinence was reported in nearly half (49.3%) of the patients with PSP [40]. Some individual items further showed significant treatment effects over time, including urinary incontinence (*P* = 0.0027), with further significance for sex (*P* = 0.049). Additionally, using MMRM measuring PSPRS test statistics for sexXTreatment interaction in week 52 also indicated significance for urinary incontinence (*P* = 0.006, Fig. 1 Table). Fig, 2C insert shows the time/sex/treatment graph for urinary incontinence, paralleling the PSPRS-28 findings.

### SEADL, and secondary endpoints, CGIC and ventricular volume

**Fig. 3:**
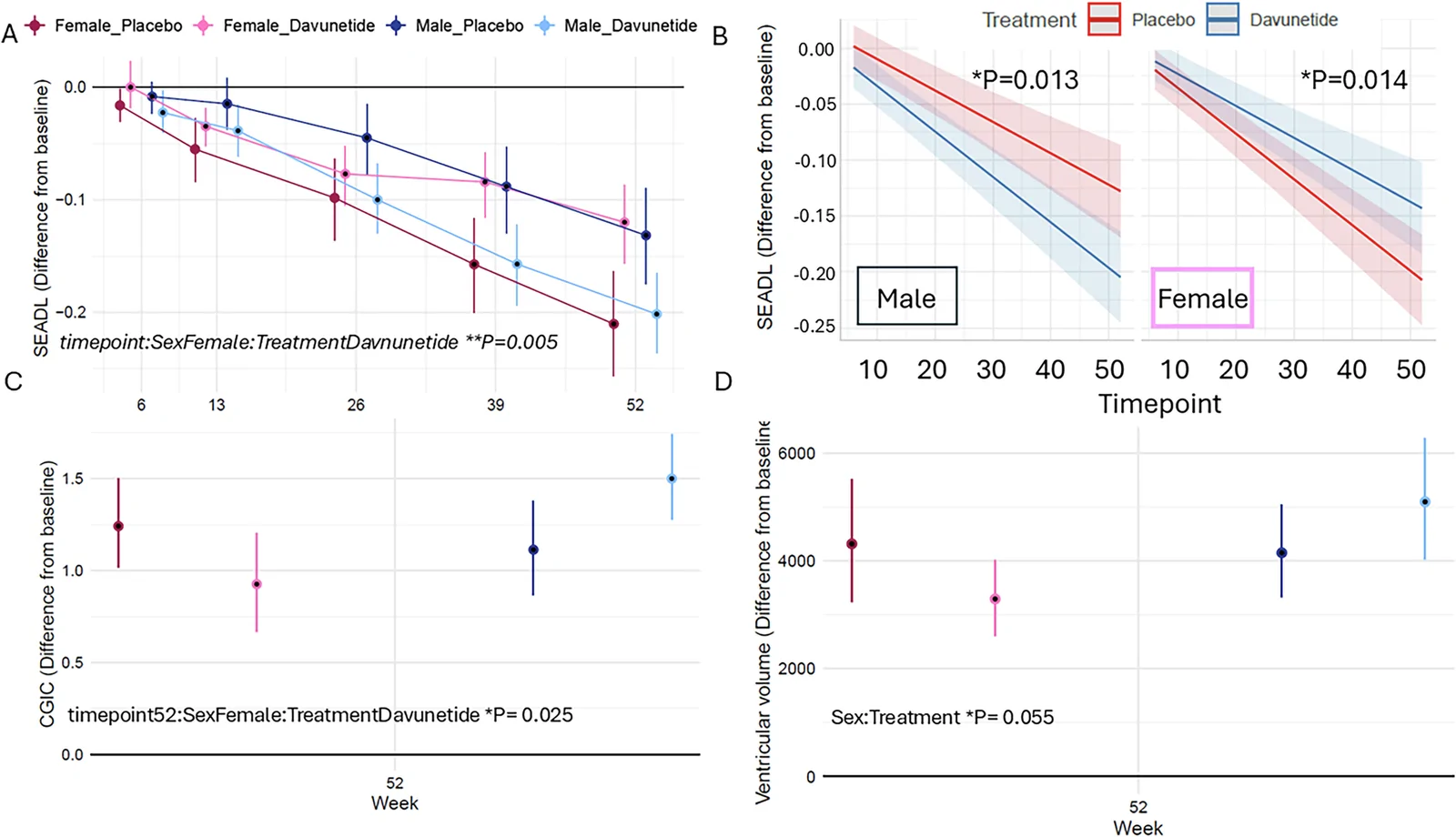
Significant Group Differences Observed in SEADL Score and Secondary Endpoints. **A** Results show a comparison of the four experimental groups for SEADL using the same parameters, populations, and calculations as Fig. 2. SEADL score difference from baseline over time per sex-treatment group is plotted by mean and confidence interval (Tables S5 and S6 show group sizes and statistical evaluations, italics depict statistical calculations by GLMM). **B** Marginal means for the SEADL score differences from baseline are shown by treatment and sex over time. **C** CGIC difference from baseline after 52 weeks of twice daily treatments, per sex-treatment group is plotted by mean and confidence interval (Tables S7 and S8 show the cohort size and statistical considerations). **D** Ventricular volume difference after 52 weeks of treatment is depicted. (Tables S7 and S8 show the cohort size and statistical considerations).

A re-assessment of the SEADL score differences [26] from baseline over time, per sex-treatment group was plotted as means and confidence interval (Fig. 3A). The precise number of individuals participating per tested group (having all PSPRS measurements per time point is indicated in Table S5), test statistics of SEADL sex differences (SexXTreatment interaction) are shown in Table S6, with GLMM further boosting the significance of our previously published result (timepoint:Sex:Treatment, *P* = *0.005*, Fig. 3A). As noticed before [26], the SEADL score showed highly significant differences already at 39 weeks of davunetide treatment with *P* = 0.007 for sex differences, *P* = 0.011 for males and *P* = 0.028, for females. These significant differences persisted in week 52, showing efficacy for davunetide in women (Fig. 3B, Table S6).

Regarding the secondary endpoints, Clinical Global Impression of Change (CGIC) is purely ordinal and measured at two timepoints. To accommodate this, we used a generalized estimating equation for ordinal responses, as implemented in the multgee package [41]. The difference from baseline over time, per sex-treatment group, plotted mean and confidence interval is shown in Fig. 3C (Table S7 indicates the number of tested individuals). Additionally, Table S8 shows ordinal GEE-regression table for CGIC indicating a timepoint52:SexFemale:TreatmentDavunetide significance of *P* = 0.025.

Regarding the ventricular volume, secondary endpoint, as this is a two-timepoint measurement, we used the more compatible type-II ANOVA. Results shown in Fig. 3D. The SexXTreatment interaction was slightly below significance, but ventricular volume increase followed the same trend as the other metrics. Davunetide females experienced the smallest growth in ventricular volume, followed by male placebo and by a large margin, female placebo and male davunetide. Table S9 shows the ventricular volume cohort size, stratified by sex and treatment, while the ANOVA Table S10 depicts the statistical analysis for ventricular volume change, Sex:Treatment *P* = 0.055.

### Sex-specific efficacy in exploratory endpoints, RBANS and executive function scores

**Fig. 4:**
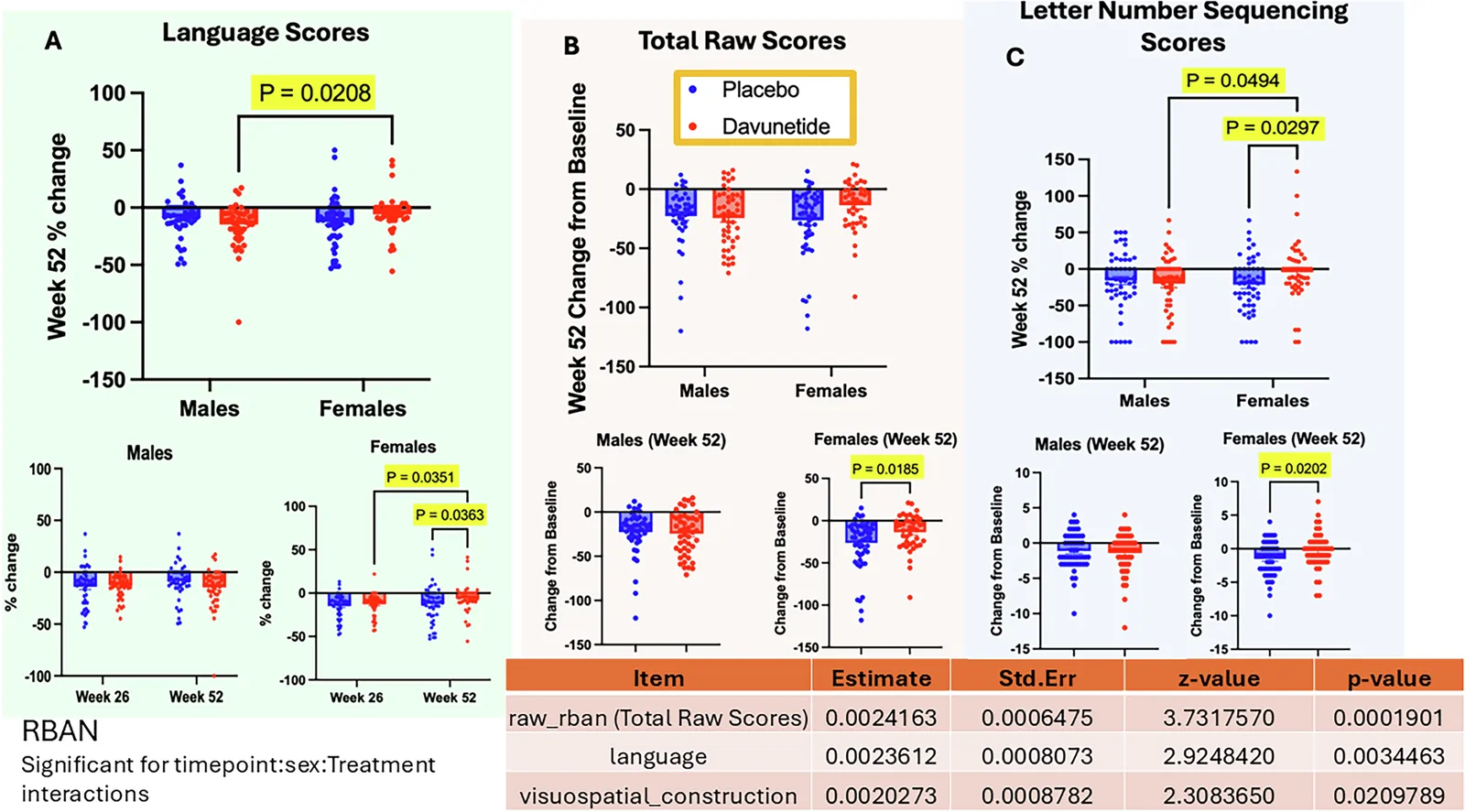
Davunetide Protects Language and Letter Number Sequencing Scores in Women. **A** Two-way ANOVA analysis of male and female Language scores percent change from baseline at Week 52 with significance seen between males and females in the davunetide experimental group (P = 0.0208), Two-way ANOVA analysis of male scores percent change from baseline compared at Weeks 26 and 52, Two-way ANOVA analysis of female scores percent change from baseline compared at Weeks 26 and 52 with significance seen between davunetide Week 26 and 52 (P = 0.0351) and between placebo and davunetide at Week 52 (P = 0.0363). **B** Two-way ANOVA analysis of male and female Total Raw scores numeric change from baseline at Week 52, Unpaired t-test analysis of male scores numeric change from baseline at Week 52 compared between placebo and davunetide experimental groups, Unpaired t-test analysis of female scores numeric change from baseline at Week 52 compared between placebo and davunetide experimental groups, with significance seen (P = 0.0185). **C** Two-way ANOVA analysis of male and female Letter Number Sequencing scores percent change from baseline at Week 52 with significance seen between males and females in the davunetide experimental group (P = 0.0494) and between female placebo and davunetide experimental groups (P = 0.0297), Unpaired t-test analysis of male scores numeric change from baseline at Week 52 compared between placebo and davunetide experimental groups, Unpaired t-test analysis of female scores numeric change from baseline at Week 52 compared between placebo and davunetide experimental groups with significance seen (P = 0.0202). Table of collected significant results GLMM is included.

Our 2023 paper [26] also briefly mentioned the significance of exploratory endpoints, which we elaborate on here. RBANS and Executive Function scores showed sex-dependent significant results, efficacy in females, in the change from baseline scores of three of the assessments: Language (Fig. 4A), Total Raw (Fig. 4B), and Letter Number Sequencing (Fig. 4C) using two-way ANOVA analysis. When examining the Week 52 percent change from baseline scores in the Language assessment, comparing males and females, a significant difference was seen between males receiving davunetide and females receiving davunetide (*P* = 0.02), a difference not seen in the placebo groups. Additionally, while no significant differences were seen in the male cohort, when comparing percent change from baseline scores for both placebo and davunetide experimental groups between Week 26 and 52, in the female cohort, significance was seen between Weeks 26 and 52 in the davunetide group (*P* = 0.035), indicating a female-specific significant improvement over time, and between the placebo and davunetide groups in Week 52 (*P* = 0.036), and implying significant improvement in Week 52 in the davunetide-treated group over the placebo-treated group (Fig. 4A).

In the Total Raw assessment, no significant differences were observed between the sexes; however, when comparing placebo- and davunetide-treated females for Week 52 numeric change from baseline scores, significance was found (*P* = 0.018), further indicating female-only davunetide-induced significant improvement (Fig. 4B).

In the Letter-Number Sequencing assessment, significance was seen when comparing the Week 52 percent change scores between males and females in the davunetide group (*P* = 0.049) and between the female placebo and davunetide groups (*P* = 0.03), further suggesting unique improvement in the davunetide-treated female cohort. This improvement was similarly seen when comparing placebo- and davunetide-treated females for Week 52 numeric change from baseline scores (*P* = 0.02), not seen in males (see Fig. 4C).

Under the same GLMM interaction term of timepoint:Sex:Treatment, we found significant results for raw RBANS (*P* = 0.0002) and language scores (*P* = 0.0003), as well as for the bulbar and gait midline domains of the PSPRS-28 (*P* < 0.05), as reported before [26]. Similarly, visuospatial construction (part of the RBANS assessment) also showed significance (Fig. 4, Table).

Looking closely at the graphs (Figs. 2 and 3) suggested an inflection in the slope after 26 weeks of twice daily davunetide treatment in females, with continuous deterioration in the placebo group, contrasting an apparent plateau in the drug-treated group, and potentially indicative of disease modification. This result was repeated for the PSPRS and the PSPRS domains, bulbar, and limb-motor (Fig. S1).

### Comorbidities reflected in evenly distributed adverse events

PSP commonly presents with comorbidities involving cardiovascular, neurological, and urological systems. Key comorbidities include hypertension, depression, diabetes mellitus type II, and infections like pneumonia (often the cause of death). Patients frequently experience falling, dysphagia, and dementia, necessitating supportive care and leading to high hospitalization rates [42].

Here, we also checked for adverse events (including potential comorbidities found in the placebo) and saw a similar distribution between study groups (Fig. S2). As delineated before, all serious adverse events in both groups were judged to be disease-related or attributable to disease progression. Most adverse events were mild to moderate and were evenly distributed between treatment groups. More participants in the davunetide group (n = 21) than in the placebo group (n = 12) had adverse events leading to study drug discontinuation, with most related to epistaxis or nasal congestion (included in the respiratory issues indicated in Fig. S2) [30].

Notably, while PSP is a syndrome which can be caused by cerebrovascular disease (e.g. 23.3% in a cohort of 128 patients), vascular PSP can be differentiated from idiopathic PSP by a higher degree of asymmetry, lower body involvement, evidence of corticospinal and pseudobulbar signs as well as the more obvious increased frequency of stroke risk factors and neuroimaging evidence of vascular disease [43]. In our case, the number of incidents of vascular adverse events was amongst the lowest detected, including about 5 events (over a 52-week-period) per tested cohort (male/female/placebo/davunetide) (Fig. S2), ruling out vascular risk factors as a major influencing factor.

### The PSP molecular target, tau shows opposite sex correlations with grasping/imitative/utilizing behavior and language

**Fig. 5:**
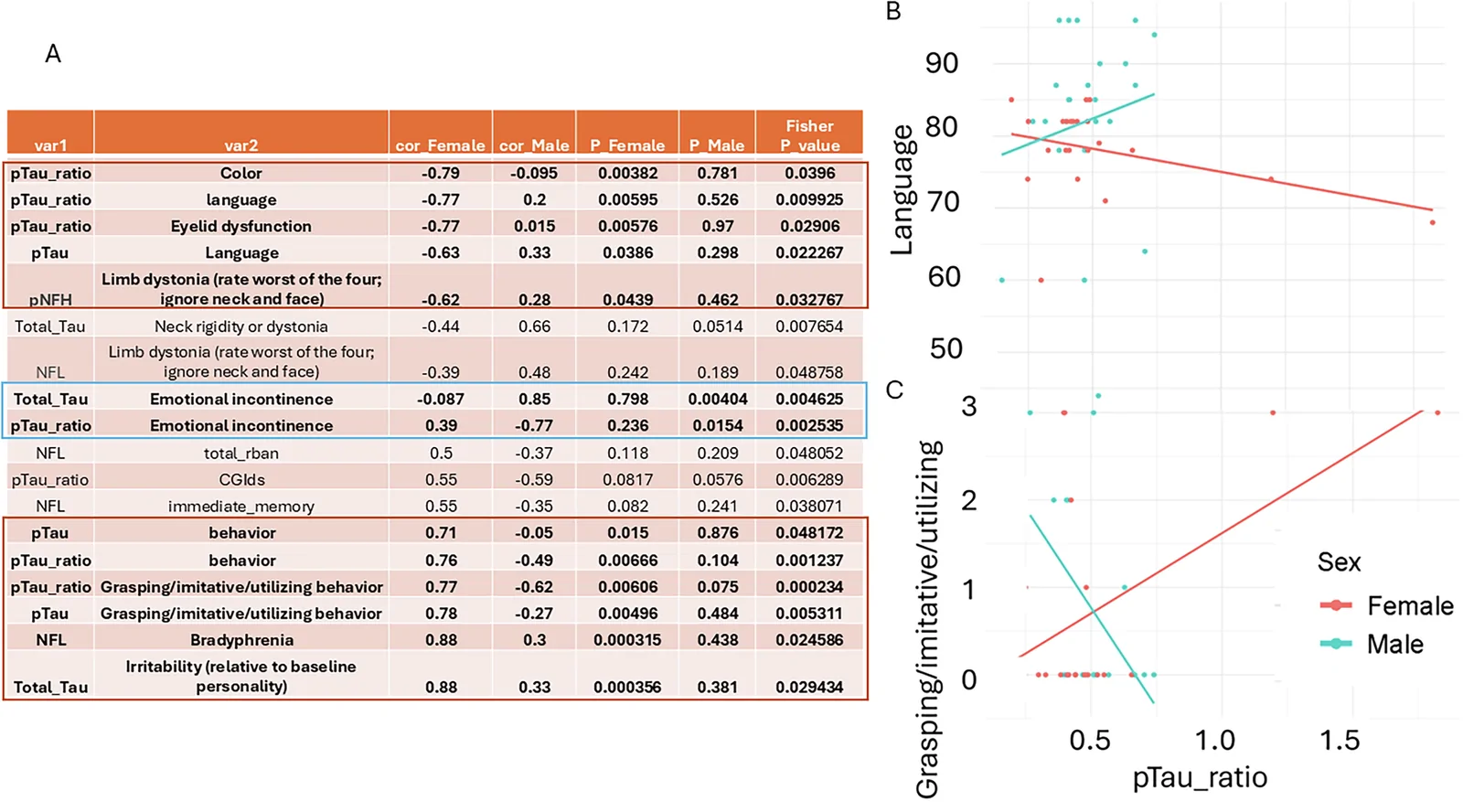
Sex-Dependent Correlations of Biomarkers (pTau, Total Tau, pNFH and NFL) with Disease Outcomes. **A** The table outlines the correlation (cor) of a variable (var) pair for each sex, followed by the significance of difference between the two (Fisher P-value) Outlined orange boxes indicate significant correlations in women, negative correlations with color trails test, and language as expected and with eyelid dysfunction and limb dystonia, which needs to be further investigated. Positive correlations are seen with PSPRS values positively indicating disease progression. Outlined blue/green box depicts men correlations with Tau and emotional incontinence. **B, C** Opposite correlations with language and grasping/imitative/utilizing behavior and pTau/total Tau (pTau_ratio), respectively, in men and women, indicative of target engagement.

In Fig. 5, we correlated all study outcome measures with Total_Tau, phosphorylated Tau 181 (pTau), pTau/Total_Tau (pTau_ratio), and neurofilament light chain (NFL) cerebrospinal fluid (CSF) concentrations, after lumbar puncture. We further correlated plasma phosphorylated neurofilament heavy chain (pNFH) with all study outcomes, showing only significant correlations. Thus, Fig. 5A, Table shows significant differences between male and female correlations (a significant Fisher P value in 18 different measurements). A closer inspection of Fig. 5A revealed that females showed 11 significant correlations, while males showed only 2, a positive correlation with total Tau and a negative correlation with pTau_ratio of emotional incontinence (blue/green square, part of the PSPRS-28, excluded from the PSPRS-10, Fig. 1). In contrast, in females the effects on total Tau and pTau in correlation with language (Fig. 5B) and grasping behaviors (Fig. 5C, also included in PSPRS-28, excluded from PSPRS-10) were aligned and indicated molecular deterioration positively correlated with PSPRS values increasing with increased Tau pathology, while augmented Tau pathology being indicative of language deterioration (Fig. 5B, C). Similarly, in women, increased NFL was correlated with bradyphrenia (slowing of thought and mental processing speed), while pNFH was negatively correlated with limb dystonia. Thus, a completely different correlation representation was found for men and women with the tested molecular biomarkers, especially those specific for tauopathy, the molecular pathology of PSP.

### Sex and treatment-specific patterns in outcome importance

**Fig. 6:**
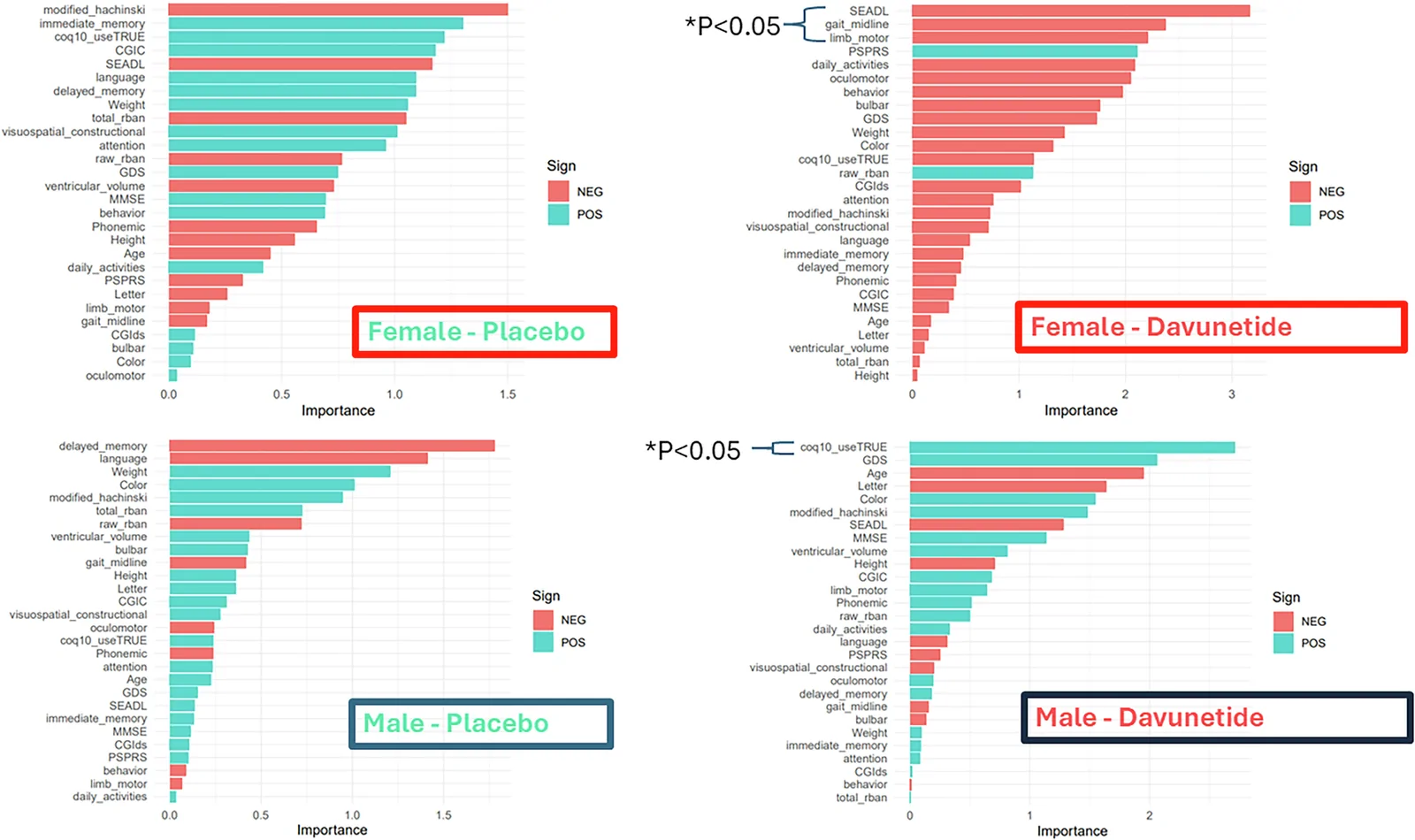
Baseline Predictors of Disease Progression per Group. Bar plots measuring the importance of individual baseline parameters in the prediction of summarized PSPRS. The importance is inferred from a generalized linear model, represented by a bar, colored according to its effect on PSPRS. A separate prediction model is shown for each sex-treatment group. Note, unlike the figures, the text includes rounded P values.

Figure 6 outlines clear sex- and treatment-specific patterns in outcome importance. In women receiving davunetide, the SEADL score (*P* = 0.007) and clinically relevant PSP domains, most notably, gait–midline, limb motor, bulbar function, and overall PSPRS, merge as the most influential variables, contrasting with women in the placebo group, where cognitive and global measures were more important. In men, davunetide did not shift importance toward PSP-specific motor domains, instead showing prominence of non-disease or baseline-related measures, supporting a female-specific therapeutic signal.

To further focus on sex differences, 4 correlation matrices were prepared, separating male and female, placebo and davunetide groups, and further focusing on PSPRS, SEADL (primary endpoints), as well as total RBANS, language, and letter (numbering sequencing) associated with cognitive functions and showing improvement only in women (above). Results clearly indicated robust differences between male and female placebo groups (Fig. S3), e.g., almost no correlations in letter (numbering) changes in females compared to several correlations to other outcomes in males, contrasting the SEADL scale, with multiple correlations in females and much fewer correlations in males (Fig. S3). In the davunetide groups, a similar difference between males and females was observed in the SEADL scale as well as the letter (numbering) (Fig. S4), while the language measurement showed correlation only with RBANS for both sexes, and with age in men only (Fig. S4). Further correlations with baseline values for the davunetide-treated patients showed a significant sex difference at week 26, when measuring SEADL change from baseline, indicating an R value of -0.432 (*P* = 2.08E-7) in men and no correlation in women, (*R* = -0.083, *P* = 0.38), while the placebo groups showed a minimal correlations (*R* = -0.293, *P* = 0.0006, men, *R* = -0.245, *P* = 0.0086, women). Together, the results implicated a sex-dependent disease course and differential effects of davunetide in men and women.

## Discussion

With the FDA preferring the recently developed PSPRS-10^31^ for measuring clinical efficacy in PSP, our post-hoc analyses utilizing the most advanced statistical assessment now revealed a highly significant treatment effect favoring women when accounting for time, sex, and treatment interactions (*P* = 0.0005). Furthermore, our current results agree with recent data strongly correlating language impairment with a faster progression in PSP-Richardson syndrome [28], while highlighting men/women differences, with davunetide being efficacious in increasing language abilities in women only, and with language deterioration significantly correlated with pTau as well as pTau/Tau in women only. Further correlating CSF pTau/Tau with PSPRS-28 item outcomes revealed the most significant women’s correlation with grasping/imitative/utilizing behavior (assessing involuntary actions). While predicting the final PSPRS score (week 52), addressing all clinical measures, we showed dramatic woman-treatment-specific patterns in outcome importance, strongly stressing the necessity for sex-specific medicine with davunetide as a lead compound. The current results are in line with our recently published data on PSP [26], showing a decrease in pTau following davunetide treatment in women only (despite a very small sample size). Strengthening this point, the considerations here with the larger population of treated and untreated and two data points, showed highly significant correlations with the study outcomes. Further corroborating data are our results assessing verbal/auditory short-term memory skills (digit span) in an amnestic mild cognitively impaired population, indicating significant protection by davunetide on this measure in females [22].

Most importantly, the overall quality of life was measured by the slower decline in major functions, which were significantly correlated between the improved PSPRS and SEADL primary outcomes in the davunetide-treated women (Fig. 6). Essential daily life independent activities including, the use of knife and fork, buttoning clothes, washing hands, and face were significantly preserved upon treatment in women (Fig. 1, *P* < 0.005). Additionally, neck rigidity was also significantly preserved, while postural stability strongly trended toward significance in the davunetide-treated women. Together with davunetide treatment in women significantly protecting language and memory scores (Fig. 4), all attested to the better quality of life following davunetide treatment in the context of pre-trial situations.

Notably, as cited above, our cohort included PSP Richardson Syndrome (PSP-RS) patients only [30]; thus, additional soluble biomarkers, like glial cell line-derived neurotrophic factor (GDNF), differentiating PSP-RS from PSP Parkinsonism Predominant (PSP-P) were not evaluated here. However, in view of new data revealing significantly increased concentrations of GDNF in the CSF among PSP-RS patients and substantially increased concentrations of GDNF in the serum in PSP-P [44], GDNF as a biomarker should be considered for future studies, further emphasizing its potential sexual dichotomy [45].

With PSP being a rare, aggressive neurodegenerative brain disorder, often confused with Parkinson’s disease (PD) due to overlapping symptoms like rigidity and slowness of movement, and given davunetide (NAP) being an active site of ADNP, it is of interest to introduce a recent finding revealing *ADNP* as a risk gene for PD [46]. Integrating machine-learning prioritization with gene- and domain-level burden testing uncovered a significant PD association in the zinc-finger domain of ADNP, including the davunetide site [46]. In agreement with these findings, dysregulation of ADNP/SIRT1 associated key histone-interacting protein transcripts was revealed in postmortem substantia nigra RNAseq data from PD compared to matched controls, similar results were seen also in postmortem AD brains, albeit in multiple brain areas [47]. Mechanistically, ADNP and SIRT1 both interact with EB1/EB3, protecting against tauopathy through the davunetide motif, SIP [47]. Contrary to AD, which is more prevalent in women [1], like PD [48], PSP is suggested to exhibit the same sex different prevalence (potentially 50% more men) [49]. (https://pmc.ncbi.nlm.nih.gov/articles/PMC12739425/).

From a genetics perspective, the H1 haplotype, which is the most prevalent in PSP [50] (see also Introduction), is also associated with PD [51] and AD (https://www.alzforum.org/mutations/mapt-haplotype-h1h2), but to a lower extent, with different genes specifically affecting each of the diseases [52]. While PSP and AD are both tauopathies involving abnormal Tau protein accumulation, those diseases fundamentally differ. PSP is a “pure” 4 R tauopathy (Tau in neurons/glial cells “tufted astrocytes”, and oligodendrocytes, no amyloid) presenting with Parkinsonism and early falls. AD is a mixed neuronal Tau/amyloid pathology (3 R/4 R Tau) causing memory-driven dementia. Despite these differences, both are neurodegenerative disorders involving hyperphosphorylated Tau, which leads to cytoskeletal disruption and nerve cell death. Furthermore, it was recently reported that about 70% of patients with PSP eventually progress to dementia, and a large proportion receive a diagnosis of dementia within the first 5 years of PSP onset, often preceding motor worsening [53], with the rate of cognitive decline differing for women and men centered on the level of baseline executive dysfunction, PSP-phenotype and age [54].

In trying to understand the basis for potential sex differences in neurodegeneration, much research has been conducted looking into how the X chromosome [55], which is linked to a variety of genetic factors that can majorly impact cognitive aging. Cognitive function and brain aging studied in female mice show how the maternal X chromosome plays a significant role in these processes. The results suggest a robust explanation as to why females, with a stronger skew towards active maternal X chromosomes, may be more susceptible to cognitive decline and potentially are more responsive to treatment [55]. Other research has shown how age-related cognitive decline and vulnerability to neurodegeneration (i.e., AD) are strongly linked to the reactivation of previously silenced X-linked genes, providing a potential pathway to female neurodegeneration resilience [56]. For example, the sodium/hydrogen exchanger 7 (NHE7), a protein crucial for maintaining pH balance and ion (Na⁺, K⁺, H⁺) levels in the Golgi apparatus and endocytic pathway, vital for proper protein processing and trafficking, encoded by the X chromosome, may influence one’s likelihood to develop AD [57].

Not just limited to neurodegeneration and AD, the X chromosome has also been shown to be strongly associated with autism spectrum disorder (ASD), with specific genes on the chromosome explaining the male prevalence seen in ASD [58]. This research suggests a chromosomal-based explanation for the observed sex differences in both neurodegeneration and other disease progression that could potentially explain the differences observed in our research.

The potential causes go beyond chromosomes. Research is further showing that menopausal hormone therapy also has a strong impact on neurodegeneration, obviously differentiating the sexes [59]. A 2025 article [60] identifies sex differences in Tau accumulation depending on the severity of atherosclerosis. Additionally, a machine learning approach to analyze sex-specific genetic connections with AD further suggested that AD may progress through different biological mechanisms in males (stress-response pathways) and females (cell-cycle pathways), highlighting the importance of sex-specific research into AD tauopathy in general and neurodegeneration, in particular [61].

It is essential to note that, although sex differences in PSP are highly significant in the results presented above, and being a pure tauopathy related to AD, these findings have been debated in previous publications. In opposition, Baba et al. (2006), as cited in the original davunetide clinical trial paper [30], suggested no sex differences in PSP, showing that the age of symptomatic onset (66.2 and 68.5 years, men and women, respectively) and disease duration (4.6 and 4.3 years, respectively) were similar in men and women. These findings contrast with recent findings from Mahale et al. (2022) [49], indicating that male presentation was significantly older (65.2 ± 7.2 years; range: 48–86 years) than female (61.9 ± 8.3 years; range: 41–80) (*P* = 0.001) and that males had significantly older age of disease onset (62.5 ± 7.3 years; range: 43–85 years) than females (57.8 ± 8.3 years; range: 40–79 years) (*P* = 0.002), with the duration of illness significantly longer in males (*P* = 0.02). One potential explanation for the contrarian findings of Baba et al. is the small sample size (68 female and 53 male patients) compared to Mahale, which had double the number of females and almost 4x the number of males.

Another potential explanation as to why sex differences were not observed by Baba may relate to estrogen, which Mahale, but not Baba, addresses. This may explain why male symptomatic onset appears similar when comparing Baba and Mahale but significantly differs in females. As mentioned above, Park et al. (2018) [27] found that estrogen replacement therapy halved PSP risk (odds ratio = 0.52; 95% confidence interval = 0.30-0.92; *P* = 0.03). This point was further discussed in a recent paper finding sex differences in clinical presentations in four repeat tauopathies [62]. It is important to note that estrogen replacement therapy was also not addressed in our cohort [26, 30].

Further findings by Mahale support our results, with shorter disease duration from onset to core PSP disease milestones such as bulbar functions, including unintelligible speech (women, 5.0 ± 1.7 years, men, 6.7 ± 3.0 years, *P* = 0.03) and severe dysphagia requiring percutaneous endoscopic gastrostomy in female PSP patients. Additionally, compared to males, female PSP patients showed shorter duration from disease onset to reach severe cognitive impairment (women, 4.6 ± 0.9 years, men, 7.0 ± 2.6 years, *P* = 0.04) [49], in agreement with our results. Takamatsu & Aiba (2021) further revealed sex differences in their results, showing reduced skeletal muscle mass versus controls, greater declines in leg mass and basal metabolism (*P* < 0.001), higher obesity rates (*P* = 0.018), and muscle loss linked to fluid balances, greater in female PSP patients than males. Their results further suggest that a high proportion of PSP patients, particularly women, experience low muscle mass [63].

As is evident, research continues to suggest the importance of sex-specific analysis in PSP. Previous studies have shown that men and women express distinct cognitive decline patterns, with women expressing a worse overall decline on the dementia rating scale construction across people less than 65 years of age, contingent on many factors [54], and that, in another cohort, significantly increased apathy was seen in men, with further sex-based differences increasing after 52 weeks of assessments [64]. It is worth noting that in the latter study, there was a marked difference between patient numbers at baseline and 52 weeks (a study patient count halving throughout the 52 weeks), and that the authors did not measure individual changes from baseline. Regardless, as apathy affects 56% of PSP patients [40], this result is of interest. Additionally, in a double-blind, placebo-controlled, 12-week/16 weeks follow-up, clinical trial studying davunetide responses in amnestic mild cognitive impairment, preceding AD (mentioned above), sex-dependent differences in cognitive response and anxiety correlations were shown, highlighting the importance of sex-specificity in tauopathy treatment [22].

Emerging mechanistic evidence further emphasizes the biological necessity of sex-specificity in PSP analysis. Firstly, being that PSP is an adult, aging neurodegenerative disease, it is important to recognize that aging shows sex-biased chromatin accessibility [65]. Additionally, increased filamin-A levels in PSP brains enhance Tau phosphorylation and insolubility through actin filament interactions [66], which further interact with androgen-regulated nuclear function, implicating sex hormones in disease modulation [67]. Lastly, the unfolded protein response (UPR) is activated in disease-affected brain regions (tauopathy) in PSP, with genetic evidence showing that this activation increases PSP risk rather than being protective, as Eukaryotic Translation Initiation Factor 2 Alpha Kinase 3 (EIF2AK3) coding variants influencing UPR activation exhibit a haplotype that is associated with increased activation and elevated PSP risk [68], and the UPR is additionally regulated by androgens and linked to muscle atrophy [69].

Tying it all together, our most recent results in an ADNP genome-edited mouse exhibiting sex-dependent developmental, vocalization, and gait impairments, as well as male accentuated tauopathy paralleled by synaptic dysfunction at the structural and functional level, indicate an underlying mechanism with completely different hippocampal male–female gene regulation. Thus, only in males, and not females, the UPR is dysregulated because of the ADNP mutation and is repaired by davunetide treatment [6]. Given that activated UPR may present a risk for PSP, this sexual dichotomy, coupled with sex-differential progression/differential correlation of disease symptoms as well as sex-dependent drug bioavailability/metabolism [15], may partly explain efficacy in women, and lack of efficacy (or potential inhibitory effects) in men.

Additionally, modeling PSP in mice, namely the rTg4510 mouse model of tauopathy, rapid deterioration in spatial learning and memory, which paralleled a significant increase in hyperphosphorylated Tau in the brain between 3 and 5.5 months of age, was seen. At 5.5 months, rTg4510 females showed significantly higher levels of hyperphosphorylated Tau than males. This increase paralleled a more severe impairment in spatial learning and memory in females as compared to transgenic males [2]. The results were corroborated by an independent investigation [4], further indicating that female rTg4510 mice displayed significantly more aggressive accumulation of pathological Tau in the brain and an increase of total Tau in CSF than their male littermates [4]. Most interestingly, studying the same mouse model [70], we discovered an early increase (3 months of age) in ADNP expression in male mice, suggesting a possible endogenous ADNP protective effect in males [70]. Together with the mechanistic finding above, these PSP model results may partially explain the observed baseline correlation with SEADL changes at 26 weeks in davunetide-treated males only (potentially expressing higher ADNP levels as also discovered in human brain postmortem analysis) [71], coupled with a slower disease deterioration in men and an overdose effect in the davunetide-treated men.

Furthermore, here, well aware of these sex differences, and translating to humans, we employed the FDA-recommended PSPRS-10 MMRM analysis, coupled with the Poisson timepoint GLMM analysis, and showed significant differences between males/females/treatment along time, with disease progression paralleling previously reported results in placebo/natural history cohorts [33], ensuing with attenuation of disease progression in davunetide-treated women. A major drug/time/sex effect was found on several disease pathologies, in agreement with our previous data showing davunetide’s strong sex-specific effects in animal models with central and peripheral muscle deficiencies, as well as cognitive impairments [5, 20, 72]. Together, our results strongly stress the necessity of sex-dependent medicine while paving the path to the clinical development of davunetide and implementation in PSP woman patients.

Therefore, a clear roadmap for future sex-stratified trials implemented at the level of the statistical analysis plan is now suggested, with evidence-based power analysis delineated in the methods section. Female-enriched sex-stratified dose optimization relying on the current study and a previous study in Pre-AD with twice daily 15 mg davunetide significantly boosting cognitive functions in a 12/16-week trial [22]. Thus, given the current results attesting for cognitive improvement of davunetide in women after 52 weeks (30 mg twice daily applications) and independent data showing specific impairment of visuospatial cognition in PSP [73], a longitudinal assessment of cognitive changes, alongside PSPRS-10 with a specific focus on independent daily activities, postural stability, bulbar/language functions in women, further correlating with SEADL outcome, is suggested. Notably, while memory issues exist in PSP, cognitive changes are often characterized more by severe apathy, personality changes, and “slowness of thought” rather than exacerbated amnestic memory loss found in AD. In this respect, male PSP patients were shown to present higher apathy scores than females [64], and apathy as well as anxiety, which is affected by davunetide [22] should be tested as well.

Regarding blood/CSF biomarkers, Tau, pTau, NFL, and pNFH should be included as performed here, as well as GDNF, as suggested above [44]. An additional biomarker to be considered is Optical Coherence Tomography (OCT), a noninvasive measure showing that the mean paramacular thickness and volume, the complex of retinal ganglion cell- and inner plexiform layer, as well as the outer nuclear layer, are reduced in PSP [74]. Importantly, ocular diseases are responsive to davunetide treatment as shown in several independent animal studies [75–77].

Pharmacokinetic considerations, as performed in our most recent study [15] will further ascertain bioavailability and compare male/female characteristics at the beginning and after a 52-week trial, with possible extension to 78 weeks.

In conclusion, the lack of effective pharmacological interventions and failure of previous trials [78] underscores the urgent need for dedicated research and therapeutic development, employing outcome measures as delineated here, while separating the sexes and showing sex-dependent significant efficacy, like davunetide in women, toward more homogeneous cohorts striving for precision medicine.

**Note added in proof**: We have just published a paper entitled "Intranasal bioavailability is estrous-cycle regulated: davunetide as a case study" [79]. This newly published study corroborates and explains mechanistic aspects of sex differences.

## Supplementary information

Supplemental Materials

## Data Availability

Full clinical data is protected due to confidentiality and may be made available from the corresponding author upon a reasonable request under a data transfer agreement.
