# Supplementary material for: Opposite molecular sex correlations in tauopathy paralleled by motor and cognitive efficacy of davunetide in women
Source: Mol Psychiatry. 2026 Jul 3;31(11):6401–12. doi: 10.1038/s41380-026-03709-x (PMC13668703; doi:10.1038/s41380-026-03709-x)
Supplement: Supplementary file 1 [file 41380_2026_3709_MOESM1_ESM.docx]

**Opposite Molecular Sex Correlations in Tauopathy Paralleled by Motor and Cognitive Efficacy of Davunetide in Women**

Guy Shapira^1^, Jason Blatt^2^, Liri S. Guz^2^, Noam Shomron^1^ and Illana Gozes^2*^

^1^Gray Faculty of Medical and Health Sciences, Sagol School of Neuroscience, Edmond J Safra Center for Bioinformatics, Tel Aviv University, Tel Aviv 6997801, Israel

^2^The Elton Laboratory for Molecular Neuroendocrinology, Gray Faculty of Medical and Health Sciences, Sagol School of Neuroscience and Adams Super Center for Brain Studies, Tel Aviv University, Tel Aviv 6997801, Israel

*Correspondence: [igozes@tauex.tau.ac.il](mailto:igozes@tauex.tau.ac.il)

**Supplementary Materials:**

**Contributing clinicians and scientists to study design and execution:**

Adam L Boxer^1^, Anthony E Lang^2^, Murray Grossman^3^, David S Knopman^4^, Bruce L Miller^5^, Lon S Schneider^6^, Rachelle S Doody^7^, Andrew Lees^8^, Lawrence I Golbe^9^, David R Williams^10^, Jean-Cristophe Corvol^11^, Albert Ludolph^12^, David Burn^13^, Stefan Lorenzl^14^, Irene Litvan^15^, Erik D Roberson^16^, Günter U Höglinger^17^, Mary Koestler^5^, Clifford R Jack Jr^18^, Viviana Van Deerlin^19^, Christopher Randolph^20^, Iryna V Lobach^5^, Hilary W Heuer^5^, Illana Gozes (corresponding author of the current post hoc analysis)^21^, Lesley Parker^22^, Steve Whitaker^23^, Joe Hirman^24^, Alistair J Stewart^25^, Michael Gold^26^, Bruce H Morimoto^27^, AND AL-108-231 Investigators^28^

Affiliations in when Boxer et al. (2014)(1) was published:

^1^Memory and Aging Center, Department of Neurology, University of California, San Francisco, CA, USA Corresponding author, electronic address: [aboxer@memory.ucsf.edu](mailto:aboxer@memory.ucsf.edu).

^2^Department of Neurology, University of Toronto, Toronto, ON, Canada

^3^Department of Neurology, University of Pennsylvania, Philadelphia, PA, USA

^4^Department of Neurology, Mayo Clinic, Rochester, MN, USA

^5^Memory and Aging Center, Department of Neurology, University of California, San Francisco, CA, USA

^6^Department of Psychiatry and the Behavioural Sciences and Department of Neurology, Keck School of Medicine of the University of Southern California, Los Angeles, CA, USA

^7^Department of Neurology, Baylor College of Medicine, Houston, TX, USA

^8^Institute of Neurology, University College London, UK

^9^Department of Neurology, Rutgers Robert Wood Johnson Medical School, New Brunswick, NJ, USA

^10^Faculty of Medicine, Monash University, Melbourne, Victoria, Australia

^11^Assistance Publique-Hôpitaux de Paris, INSERM, CIC1422 and UMRS1027, Sorbonne Universités, Université Pierre et Marie Curie, Paris, France; Department of Neurology, Pitié-Salpêtrière Hospital, Paris, France

^12^Department of Neurology, University Hospital, Ulm, Germany

^13^Institute for Ageing and Health, Newcastle University, Newcastle, UK

^14^Interdisciplinary Center for Palliative Medicine, Munich University Hospital-Klinikum Grosshadern, Munich, Germany

^15^Department of Neurology, University of California, San Diego, CA, USA

^16^Department of Neurology, University of Alabama, Birmingham, AL, USA

^17^Department of Translational Neurodegeneration, Technical University Munich, Munich, Germany.

^18^Department of Radiology, Mayo Clinic, Rochester, MN, USA

^19^Department of Neurology and Pathology and Laboratory Medicine, Perelman School of Medicine, University of Pennsylvania, Philadelphia, PA, USA

^20^Department of Neurology, Loyola University School of Medicine, Chicago, IL, USA

^21^Department of Human Molecular Genetics and Biochemistry, Sackler Faculty of Medicine and Sagol School of Neuroscience, Adams Super Center for Brain Studies, Tel Aviv University, Tel Aviv, Israel

^22^Tekmira Pharmaceuticals, Burnaby, BC, Canada

^23^Omeros, Seattle, WA, USA

^24^Pacific Northwest Statistical Consulting, Woodinville, WA, USA

^25^Paladin Laboratories, St Laurent, QC, Canada

^26^UCB BioSciences, Research Triangle Park, NC, USA

^27^Celerion, Lincoln, NE, USA

^28^AL-108-231 Investigators: David Williams, Anne Louise Lafontaine, Connie Marras, Mandar Jog, Michael Panisset, Jean-Christophe Corvol, Jean-Philippe Azulay, Philippe Couratier, Brit Mollenhauer, Stefan Lorenzl, Albert Ludolph, Reiner Benecke, Günter Höglinger, Axel Lipp, Heinz Reichmann, Dirk Woitalla, Dennis Chan, Adam Zermansky, David Burn, Adam Boxer, Erik Roberson, Lawrence Honig, Edward Zamrini, Rajesh Pahwa, Yvette Bordelon, Erika Driver-Dunkley, Stephanie Lessig, Mark Lew, Kyle Womack, Brad Boeve, Joseph Ferrara, Argye Hillis, Daniel Kaufer, Rajeev Kumar, Tao Xie, Steven Gunzler, Theresa Zesiewicz, Praveen Dayalu, Lawrence Golbe, Murray Grossman, Joseph Jancovic, Scott McGinnis, Anthony Santiago, Paul Tuite, Stuart Isaacson, Julie Leegwater-Kim, Irene Litvan

**Supplemental Tables:**

Table S1: PSPRS Cohort size, stratified by sex and treatment

| PSPRS-10 | Male | | Female | |
| --- | --- | --- | --- | --- |
| Week | Placebo | Davunetide | Placebo | Davunetide |
| 0 | 60 | 56 | 56 | 54 |
| 6 | 60 | 56 | 55 | 53 |
| 13 | 56 | 52 | 56 | 53 |
| 26 | 46 | 48 | 49 | 49 |
| 39 | 42 | 44 | 49 | 47 |
| 52 | 40 | 40 | 48 | 46 |

Table S2: Test statistics of summarized PSPRS10 sex differences (SexXTreatment interaction) as well as the individual sexes

| PSPRS-10 | Sex difference | | Male | | Female | |
| --- | --- | --- | --- | --- | --- | --- |
| Week | stat | P | stat | P | stat | P |
| 13 | 2.3 | 0.02 | -1.1 | 0.27 | 0.52 | 0.6 |
| 26 | -0.33 | 0.75 | 1.18 | 0.24 | -0.25 | 0.8 |
| 39 | -1.5 | 0.14 | 1.78 | 0.08 | -1.18 | 0.24 |
| 52 | -2.59 | 0.01 | 2.26 | 0.03 | -2.09 | 0.04 |

Table S3: PSPRS-28 Sum Cohort size, stratified by sex and treatment

| PSPRS-10 | Male | | Female | |
| --- | --- | --- | --- | --- |
| Week | Placebo | Davunetide | Placebo | Davunetide |
| 0 | 62 | 63 | 70 | 71 |
| 6 | 61 | 63 | 69 | 70 |
| 13 | 62 | 63 | 70 | 69 |
| 26 | 61 | 62 | 62 | 61 |
| 39 | 61 | 62 | 61 | 57 |
| 52 | 62 | 62 | 60 | 55 |

Table S4: Test statistics of summarized PSPRS-28 sex differences (SexXTreatment interaction) as well as the individual sexes

| PSPRS-10 | Sex difference | | Male | | Female | |
| --- | --- | --- | --- | --- | --- | --- |
| Week | stat | P | stat | P | stat | P |
| 13 | -0.621 | 0.535 | 1.385 | 0.167 | -0.09 | 0.929 |
| 26 | -2.103 | 0.036 | 2.221 | 0.027 | -1.02 | 0.309 |
| 39 | -1.771 | 0.078 | 2.012 | 0.045 | -0.903 | 0.367 |
| 52 | -2.959 | 0.003 | 2.555 | 0.011 | -1.984 | 0.048 |

Table S5: SEADL Cohort size, stratified by sex and treatment

| SEADL | Male | | Female | |
| --- | --- | --- | --- | --- |
| Week | Placebo | Davunetide | Placebo | Davunetide |
| 0 | 61 | 63 | 70 | 71 |
| 6 | 60 | 62 | 69 | 69 |
| 13 | 61 | 62 | 70 | 69 |
| 26 | 60 | 63 | 62 | 61 |
| 39 | 60 | 63 | 61 | 57 |
| 52 | 60 | 63 | 58 | 55 |

Table S6: Test statistics of SEADL sex differences (SexXTreatment interaction) as well as the individual sexes

| SEADL | Sex difference | | Male | | Female | |
| --- | --- | --- | --- | --- | --- | --- |
| Week | stat | P | stat | P | stat | P |
| 13 | 0.618 | 0.537 | -1.451 | 0.148 | 0.984 | 0.326 |
| 26 | 1.331 | 0.184 | -2.377 | 0.018 | 0.663 | 0.508 |
| 39 | 2.708 | 0.007 | -2.574 | 0.011 | 2.206 | 0.028 |
| 52 | 2.914 | 0.004 | -2.488 | 0.014 | 2.506 | 0.013 |

Table S7: CGIC Cohort size, stratified by sex and treatment

|  | Male | | Female | |
| --- | --- | --- | --- | --- |
| Week | Placebo | Davunetide | Placebo | Davunetide |
| 0 | 53 | 54 | 58 | 54 |
| 52 | 53 | 54 | 58 | 54 |

Table S8: Ordinal GEE-regression table for CGIC

| Coefficient | Estimate | san.se | san.z | pvalue |
| --- | --- | --- | --- | --- |
| beta10 | -5.41702 | 1.19256 | -4.54234 | 0.00001 |
| beta20 | -2.12850 | 0.37132 | -5.73218 | 0.00000 |
| beta30 | 1.27649 | 0.32577 | 3.91834 | 0.00009 |
| beta40 | 3.34160 | 0.34390 | 9.71672 | 0.00000 |
| beta50 | 5.36408 | 0.40783 | 13.15267 | 0.00000 |
| timepoint52 | -2.78700 | 0.36459 | -7.64420 | 0.00000 |
| SexFemale | 0.07926 | 0.38590 | 0.20538 | 0.83728 |
| TreatmentDavunetide | -0.60111 | 0.41431 | -1.45086 | 0.14682 |
| timepoint52:SexFemale | -0.32109 | 0.44463 | -0.72215 | 0.47020 |
| timepoint52:TreatmentDavunetide | -0.71262 | 0.46782 | -1.52326 | 0.12769 |
| SexFemale:TreatmentDavunetide | 0.41641 | 0.52943 | 0.78651 | 0.43157 |
| timepoint52:SexFemale:TreatmentDavunetide | 1.44447 | 0.64285 | 2.24698 | 0.02464 |

Table S9: Ventricular volume Cohort size, stratified by sex and treatment

|  | Male | | Female | |
| --- | --- | --- | --- | --- |
| Week | Placebo | Davunetide | Placebo | Davunetide |
| 0 | 55 | 55 | 48 | 51 |
| 52 | 55 | 55 | 48 | 51 |

Table S10: ANOVA table for ventricular volume change

| Coefficient | stat | pvalue |
| --- | --- | --- |
| Sex | 2.677000 | 0.103 |
| Treatment | 0.000755 | 0.978 |
| Sex:Treatment | 3.727000 | 0.055 |

**Supplemental Figures:**

**Fig. S1. Analyses with an inflection point at 26 weeks: placebo - continuous deterioration, davunetide – protection as measured by the slope:**

For each metric (using R), a linear mixed model was constructed separately for before and after week 26 (including), formulated as such: value ~ week + (1|subject). The values are absolute, and the week parameter for the "from week 26 onwards" model was decreased by 26 (so that the first datapoint would be considered week 0). Here, the traditional PSPRS-28 was reanalyzed, including the bulbar and limb motor domains, and SEADL (2).

**Fig. S2. Instances of Adverse Events:**

The Table shows the number of participants separated into four groups, by sex and treatment. The graph depicts the number of instances of adverse events over the course of the clinical trial (Y axes) separated by sex and treatment (X axes).

|  | **Sample size (n)** | Davunetide | Placebo |  |
| --- | --- | --- | --- | --- |
|  | Male (n = 152) | 74 | 78 |  |
|  | Female (n = 141) | 71 | 70 |  |
|  |  |  |  |  |

Adverse Events by Sex and Treatment

|  | Male Davunetide | Male Placebo | Female Davunetide | Female Placebo |
| --- | --- | --- | --- | --- |
| Cardiac Disorders | 1 | 3 | 3 | 0 |
| Respiratory Issues | 39 | 32 | 43 | 30 |
| Blood and lymphatic system disorders | 2 | 3 | 3 | 4 |
| Eye disorders | 15 | 10 | 15 | 10 |
| GI disorders | 22 | 29 | 15 | 19 |
| Pain | 18 | 22 | 14 | 21 |
| Psychiatric Disorders | 18 | 26 | 13 | 21 |
| Skin Issues | 25 | 26 | 18 | 19 |
| Vascular Disorders | 6 | 4 | 4 | 6 |
| Tissue Disorders | 25 | 30 | 21 | 26 |

**Fig. S3,4. Cognitive Measures Significantly Correlate and Show Sex Differences for the Davunetide Groups:**

Significance between all PSPRS, RBANS, and various related cognitive scores mapped for placebo (Fig. S3) and davunetide females and males (Fig. S4). Stronger blue indicates stronger significance, and stronger red indicates weakest significance. Note total SEADL and PSPRS change correlated with Total RBAN, Language, and Letter Number Sequencing scores (boxed rows/columns).

**References:**

1.	Boxer AL, Lang AE, Grossman M, Knopman DS, Miller BL, Schneider LS, et al. Davunetide in patients with progressive supranuclear palsy: a randomised, double-blind, placebo-controlled phase 2/3 trial. *Lancet Neurol.* 2014;13(7):676-85.

2.	Gozes I, Shapira G, Lobyntseva A, and Shomron N. Unexpected gender differences in progressive supranuclear palsy reveal efficacy for davunetide in women. *Transl Psychiatry.* 2023;13(1):319.
